# The Comparison of Environmental Effects on Michelson and Fabry-Perot Interferometers Utilized for the Displacement Measurement

**DOI:** 10.3390/s100402577

**Published:** 2010-03-24

**Authors:** Yung-Cheng Wang, Lih-Horng Shyu, Chung-Ping Chang

**Affiliations:** 1 Institute of Mechanical Engineering, National Yunlin University of Science and Technology, Douliou, Yunlin County 640, Taiwan; E-Mail: g9611754@yuntech.edu.tw; 2 Institute of Mechanical and Electro-Mechanical Engineering, No.64, Wunhua Rd., Huwei Township, Yunlin County 632, Taiwan; E-Mail: lhshyu@nfu.edu.tw

**Keywords:** environmental effects, common optical path, Fabry-Perot interferometer, displacement measurement

## Abstract

The optical structure of general commercial interferometers, e.g., the Michelson interferometers, is based on a non-common optical path. Such interferometers suffer from environmental effects because of the different phase changes induced in different optical paths and consequently the measurement precision will be significantly influenced by tiny variations of the environmental conditions. Fabry-Perot interferometers, which feature common optical paths, are insensitive to environmental disturbances. That would be advantageous for precision displacement measurements under ordinary environmental conditions. To verify and analyze this influence, displacement measurements with the two types of interferometers, *i.e.*, a self-fabricated Fabry-Perot interferometer and a commercial Michelson interferometer, have been performed and compared under various environmental disturbance scenarios. Under several test conditions, the self-fabricated Fabry-Perot interferometer was obviously less sensitive to environmental disturbances than a commercial Michelson interferometer. Experimental results have shown that induced errors from environmental disturbances in a Fabry-Perot interferometer are one fifth of those in a Michelson interferometer. This has proved that an interferometer with the common optical path structure will be much more independent of environmental disturbances than those with a non-common optical path structure. It would be beneficial for the solution of interferometers utilized for precision displacement measurements in ordinary measurement environments.

## 1. Introduction

The interferometer has been widely applied to metrology since the laser has a long coherence length. The major advantage of interferometers is their coexisting characteristics of large measuring range and high resolution, but on the other hand, most interferometers are sensitive to environmental disturbances. The Fabry-Perot interferometer, consisting of two parallel planar mirrors is a kind of interferometer with the common optical path. Its interference beams are reflected and transmitted in the optical cavity forwards and backwards, so that the variation of the optical path due to the environmental effect will cancel out. For this reason, displacement measurements by the optical structure of a Fabry-Perot interferometer can be independent of environmental disturbances and it may be concluded that interferometers with a common optical path [1,2] are more resistant to environmental disturbances. In order to investigate the environmental influence, comparison measurements have been performed under some test environmental conditions between a Fabry-Perot interferometer and a commercial Michelson interferometer, which is a kind of commercial interferometer widely utilized for displacement measurements. Experimental results will be analyzed and summarized.

## 2. Measurement Principle and Theory

### 2.1. Principle of the Michelson Interferometer

**Figure 1.**
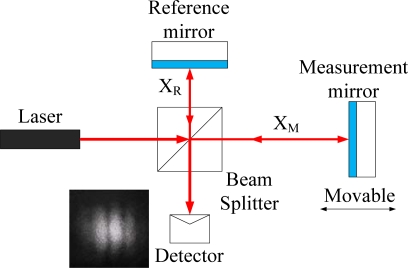
Michelson interferometer.

The fundamental structure of a Michelson interferometer [3,4] is shown in Figure 1. The incident laser beam (I_0_) is divided into two laser beams after passing through the beam splitter. They are the measurement beam and reference beam which propagate in different paths (x_M_, x_R_). After being reflected by the measurement and reference mirror, they travel back to the beam splitter and interfere. From the formula derived by following Equations (1–3), the intensity I can be expressed as Equation (5), where (E_R_: electric field of reference beam, E_M_: electric field of measurement beam, I: intensity of interference beam, λ_R_: wavelength of reference beam, λ_M_: wavelength of measurement beam):

(1) $$E_{R}=A_{R}\cdot e^{i(\omega t+k_{R}x_{R})}$$

(2) $$E_{M}=A_{M}\cdot e^{i(\omega t+k_{M}x_{M})}$$

(3) $$I=(E_{R}+E_{M})\cdot (E_{R}+E_{M})*$$

(4) $$I_{R}=I_{M}=\frac{I_{0}}{4}$$

(5) $$I=\frac{I_{0}}{2}(1-\;\text{cos}\;\delta_{1})$$

(6) $$\delta_{1}=k_{R}\cdot X_{R}-k_{M}\cdot X_{M}=2\pi \cdot (\frac{X_{R}}{\lambda_{R}}-\frac{X_{M}}{\lambda_{M}})$$

Under the ideal situation the phase difference (δ_1_) has been only induced from the displacement of the measurement mirror. But actually, not only the displacement of the measurement mirror but also air disturbance, mechanism vibration or changes of refraction index between the measurement and reference arm will cause an optical path length difference which will lead to changes of *δ*_1_. For this reason, the phase difference (*δ*_1_) is dependent on both the displacement of the measurement mirror and the variation due to environmental effects. The measurement accuracy will be disturbed because of environmental perturbations.

### 2.2. Principle of Fabry-Perot Interferometer

**Figure 2.**
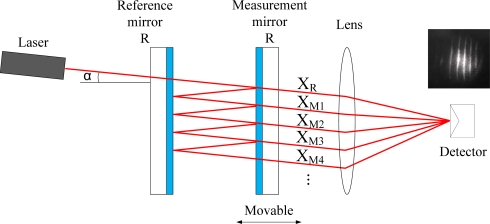
Fabry-Perot interferometer.

The typical structure of a Fabry-Perot interferometer [3,5,6] is shown in Figure 2. The incident beam (I_0_) with the tiny incident angle (α) spreads into the optical cavity which is composed of the measurement and reference mirrors. In the optical cavity, laser beams travel forwards and backwards and are divided into numerous transmitted beams. The electric field equation of each transmitted beam can be described by Formula (9).

(7) $$E_{R}=A_{0}\sqrt{1-R}\cdot e^{i(\omega t+\text{kx})}$$

(8) $$E_{M}=\sum_{n=1}^{\infty }E_{\text{Mn}}=\sum_{n=1}^{\infty }A_{0}\sqrt{1-R}\cdot R^{n}\cdot e^{i(\omega t+\text{kx}+2n\delta )}$$

(9) $$E=E_{R}+E_{M}=E_{R}+E_{M1}+E_{M2}+E_{M3}+\cdots +E_{M\infty }$$

After the transmitted beams overlap, the interferometric electric field equation can be expressed as follows:

(10) $$E=\text{Re}\left\{e^{i(\omega t+\text{kx})}\cdot (1-R)\cdot A_{0}\cdot e^{-i\delta_{2}}\cdot \frac{1}{1-R\cdot e^{i\delta_{2}}}\right\}$$

The intensity distribution of the interference beam can be deduced from the Equations (10) and (11), and represented by the following Formula (12):

(11) I=E⋅E*

(12) $$I=I_{0}\frac{{\left(1-R\right)}^{2}}{1+R^{2}-2\cdot R\cdot \;\text{cos}\;\delta_{2}}$$

where *δ*_2_ = (2·d·k)/cosα (if α∼0, *δ*_2_ = 4·π·(d/λ)) and d denotes the displacement of the measurement mirror.

### 2.3. Theoretical comparison

In Equation (6) δ_1_ is determined from the optical path difference between the measurement and reference arm. In a general situation, each wavelength in the different arms needs to be calculated for wavelength compensation. In commercialized interferometers it is difficult to satisfy the above requirement, hence only a unified compensation for the whole environment is performed. That will lead to measurement errors for ultra high precision measurements, unless displacement measurements have been accomplished under strict control of environmental conditions. In a regular measurement environment, the interferometric phase difference will be not only affected by the displacement of the measurement mirror but also by the wavelengths, which vary in different arm due to different environmental effects.

In a Fabry-Perot interferometer, δ_2_ is also affected by the displacement of the measurement mirror d and the wavelengths, but the wavelength variations are the same for all laser beams because of identical environmental conditions. Since all laser beams can be considered to possess the same wavelength, an identified compensation of the wavelength is acceptable.

## 3. Experimental Structure

**Figure 3.**
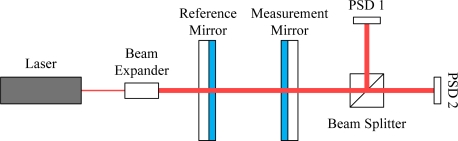
Structure of the self-developed Fabry-Perot interferometer.

**Figure 4.**
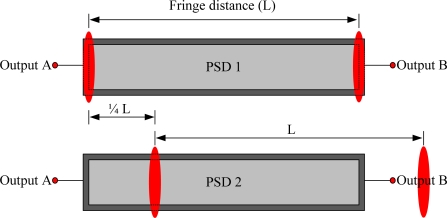
PSD1/2 and fringe distributions.

The self-developed Fabry-Perot interferometer (laser wavelength: 632.9907 nm, frequency stabilization: 3 × 10^−7^) structure is shown in Figure 3. By regulating the tilt angle of the measurement mirror, the fringe distance will be equal to the sensing width of the position sensitive detector (PSD, S3931) [7]. As shown in Figure 4, two PSDs are so arranged to detect interferometric signals with the phase shift of the quadrature periode, such that two orthogonal signals with the phase difference of 90° can be utilized for displacement measurement of the measurement mirror.

**Figure 5.**
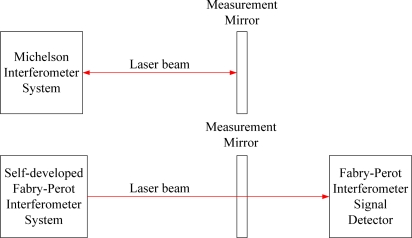
Schematic of the experimental structure for repeatability measurements.

Figure 5 is the schematic of the experimental set-up. The upper part shows a Michelson interferometer (laser wavelength: 632.8 nm, frequency stabilization: 3 × 10^−7^) with the measurement mirror. The lower one shows the self-developed Fabry-Perot interferometer, the measurement mirror and the optical detector. Some different environmental conditions e.g. regular and isolated conditions, airflow disturbance and temperature variation have been manipulated to observe their effects on both interferometers. Static measurements on a fixed position for interferometers were performed. From the measurement results of each measurement task, the concerning repeatability has been analyzed and clarified. For each measurement conditions, measurement task has been repeated 10 times. The presented measurement quantity of each measurement results from one thousand sample data and the sampling rate is 10 Hz. The measuring time is less than 30 minutes and all repeatability results will be demonstrated.

## 4. Results and Analysis

### 4.1. Regular conditions

**Figure 6.**
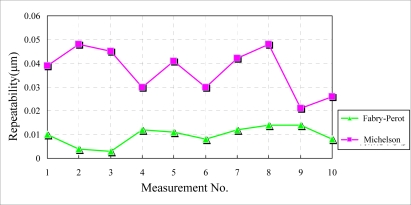
Experimental results under regular conditions.

The experimental results under the regular condition are shown in Figure 6. The environmental influence on the Fabry-Perot interferometer is obvious much weaker than that on the Michelson interferometer. The repeatability of the Fabry-Perot interferometer is about one third of that of the Michelson interferometer.

### 4.2. Isolated condition

In order to compare the measuring performance of both interferometers, a more stable environmental condition, *i.e.*, the isolated condition has been configured. An acrylic box is used to cover both interferometers. The experimental field is airtight and the environmental variation can be minimized. Under this measuring condition, the repeatabilities of both interferometers are improved because of the stable environmental conditions. The maximal measurement dispersion of the Fabry-Perot interferometer is only about 20% of that of the Michelson interferometer. It’s evident that the environmental influence on the Fabry-Perot interferometer is indistinct under regular as well as isolated conditions.

### 4.3 Airflow disturbance

**Figure 9.**
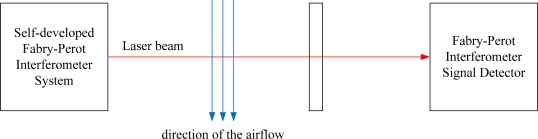
Direction of the airflow.

For investigating the effect of airflow disturbance, a flow speed of 3.2 m/s has been applied intermittently to both interferometers with an electric fan. The direction of the airflow is shown in Figure 9. The disturbance effect on the Michelson interferometer is much distincter than that on the Fabry-Perot interferometer. The repeatability of the Michelson interferometer is nearly four times more than that of the Fabry-Perot interferometer.

### 4.4. Temperature variation

**Figure 10.**
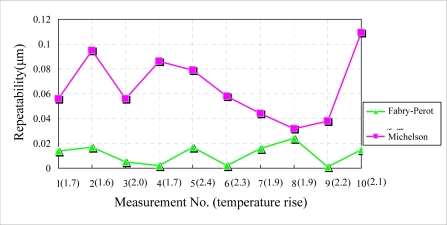
Experimental results under temperature variation.

In this experiment, the measurement field is covered by the acrylic box and a heating wire is utilized to stimulate a temperature rise around the interferometers. To detect temperatures, the temperature sensor (PT100) is placed near the interferometers to measure the temperature during the experiment. Experimental results are shown in Figure 10. The repeatability dispersion of the Michelson interferometer is relatively large because the interferometer is more sensitive to temperature effects. The maximal repeatability of the Fabry-Perot interferometer is about one fifth of that of the Michelson interferometer. This result is similar to the experimental result under airflow disturbance. In this situation the Fabry- Perot interferometer is also more stable than the Michelson interferometer.

### 4.5. Analysis of measurement results

According to the experimental results, the RMS deviations and mean values have been calculated which are tabulated in the list shown in Table 1. Under each test condition, the environmental effect on the Fabry-Perot interferometer is less than that on the Michelson interferometer. In comparison with Fabry-Perot Interferometer (F-P), the mean values of the Michelson interferometer (M) have increased about fourfold.

## 5. Conclusions

The theoretical and experimental results have shown that environmental effects have more and definitive influence on the measurement precision of commercial interferometers with Michelson structure than that of Fabry-Perot interferometers. Especially if there are temperature changes or air disturbances in measurement field, the phenomenon will be more evident. It has also proved that Fabry-Perot interferometers are more apt to resist environmental disturbances than general Michelson interferometers, because of their common optical path structure. Relatively, approximately 80% of the environmental effect on measurement error can be reduced by the use of the Fabry-Perot interferometer. For industrial applications under regular conditions, a system with a Fabry-Perot interferometer would be a robust and potential tool for displacement measurements. In this investigation it has been proven that under some test conditions the Fabry-Perot interferometer (common optical path) will be more insensitive to environmental disturbances than the Michelson interferometer (non-common optical path), but there are still will be some error sources which need to be thoughtfully verified, e.g. other influence on the measuring repeatability or experiments with different optical path lengths. These will the subject of planned future research.

## Figures and Tables

**Figure 7.**
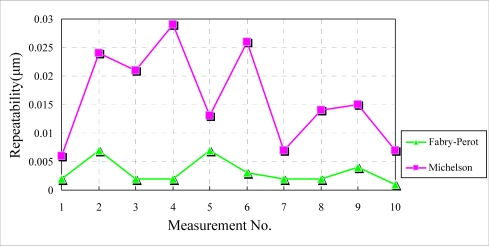
Experimental results under isolated conditions.

**Figure 8.**
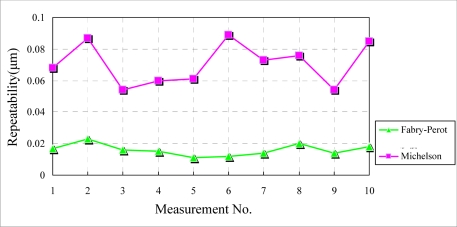
Experimental results under airflow disturbance (flow speed: 3.2 m/s).

**Table 1.** The RMS deviations and the mean values.

	RMS deviation (μm)		mean (μm)	
	F-P	M	F-P	M
Regular condition	0.0038	0.0096	0.010	0.037
Isolated condition	0.0021	0.0084	0.003	0.016
Airflow disturbance	0.0037	0.0134	0.016	0.071
Temperature variation	0.0085	0.0257	0.011	0.065
